# OSKM-mediated reversible reprogramming of cardiomyocytes regenerates injured myocardium

**DOI:** 10.1186/s13619-021-00106-3

**Published:** 2022-01-17

**Authors:** Gregory Farber, Jiandong Liu, Li Qian

**Affiliations:** Department of Pathology and Laboratory Medicine, McAllister Heart Institute, University of North Carolina, Chapel Hill, NC 27599 USA

## Abstract

Cellular reprogramming has rapidly become a promising methodology to generate new cardiomyocytes from non-cardiomyocyte cell types. Using the transient expression of OSKM factors, Chen et al. demonstrate a unique reprogramming strategy involving the modulation of the resident adult cardiomyocyte identity to an immature proliferative state (Science 373:1537–40, 2021). This OSKM-mediated reversion results in the adoption by adult murine cardiomyocytes of a transcriptional profile similar to cardiomyocytes found in developing hearts, as well as increased proliferative capacity of these reprogrammed cardiomyocytes compared to mature cardiomyocytes. Furthermore, this novel approach enhances the regeneration of adult murine hearts post-myocardial injury. Although concerns and questions remain, the encouraging results of this study advance the field of cardiac regeneration by providing a new technique to generate cardiomyocytes as well as insights into cardiomyocyte dedifferentiation and its relation to proliferation.

## Main text

The high global prevalence of cardiac injury and disease has made finding new sources for cardiomyocytes an ever-evolving area of research. Numerous approaches to repair injured cardiac tissue have been developed and range from bioengineering-focused therapies, to stem cell-based strategies, to direct manipulation of cell signaling pathways. The ultimate goal for these methodologies is to generate new cardiomyocytes and/or replace damaged ones with the aim of improving heart function. Direct and indirect (via iPSC route) reprogramming-based methods are attractive options for generating new cardiomyocytes from non-cardiomyocyte cell types and have shown promise as potential therapeutic tools for heart regeneration (Ieda et. al 2010; Qian et. al 2012; Shiba et al. 2016; Song et al. 2012; Wang et al. 2021). However, the excitement concerning these conversion techniques is tempered by the difficulty of creating fully functional and mature cardiomyocytes in vitro, as these methods generally yield cardiomyocytes of a relative immature phenotype in a dish. Utilizing native cardiomyocytes to create cardiomyocytes has always been a tantalizing proposal because of the potential to generate a cell type that is either identical or very similar to the desired adult cardiomyocyte. Furthermore, the well-characterized regenerative capacity of neonatal cardiomyocytes in murine injury models highlights the underlying potential for adult cardiomyocytes to generate new cardiomyocytes if they can re-acquire their regenerative capabilities that are loss during cardiac maturation.

In a recent paper by Chen et. al, the authors explore the possibility of reverting murine adult cardiomyocytes into a more immature state by forced-expression of OSKM factors (Oct4, Sox2, Klf4, and c-Myc) in murine cardiomyocytes, and investigate whether this reversion process allows resident cardiomyocytes to proliferate post-myocardial infarction (Chen et. al 2021). This de-differentiation reprogramming strategy resulted in decreased scar formation post-myocardial infarction, and demonstrated the potential for improving adult cardiomyocyte proliferative capacity by changing their maturation state through transient expression of OSKM in cardiomyocytes. The authors methodically evaluated how to best utilize this reprogramming strategy post-myocardial injury as well as its impact on mice of various ages. Bioinformatical analyses demonstrated that the reprogrammed identity of the affected cardiomyocytes are similar to cardiomyocytes found in developing murine hearts. However, this reprogramming approach faces similar hurdles as other methods, such as reprogramming heterogeneity and the question of what should be the final desired cardiomyocyte identity after reprogramming in order to achieve both the creation of new cardiomyocytes as well as functional improvement after cardiac injury.

Investigation of the different intermediary states resulting from OSKM factor-mediated reversible reprogramming would provide exciting insights into the molecular mechanisms behind this de-differentiation process as well as how the OSKM factors guide cardiomyocytes to regain their proliferation capabilities. Single cell genomics has allowed for the visualization of the heterogeneity of reprogrammed cells in other reprogramming methodologies and has shown the presence of multiple intermediary states between the starting cell type and final identity (Stone et al. 2019; Zhou et al. 2019). In the case of this reversible reprogramming, there is potential to see cardiomyocytes in various states of dedifferentiation, ranging from fully de-differentiated pluripotent cells to unaffected cardiomyocytes. Further evaluation could determine whether certain intermediary states may be more beneficial to cardiac regeneration than others. As shown in other reprogramming-related studies, unsuccessful reprogramming or the creation of undesired cell types can lead to negative functional consequences such as arrythmias or improperly formed muscle tissue. Single cell genomics would also allow for more detailed analysis into how closely OSKM-reprogrammed cardiomyocytes revert back to their original identity and would potentially highlight ways to improve this method.

This study also raises an interesting question on what should be the desired end product of cardiomyocyte reprogramming for cardiac regeneration. While other reprogramming strategies seek to create mature cardiomyocytes to replace damaged ones, this study seeks to obtain an earlier stage of cardiomyocyte identity which may result in increased proliferation potential at the expense of losing key functional attributes. This may explain why this technique yields new cardiomyocytes but does not result in significant improvement of cardiac function after myocardial infarction. What is necessary and not for a reprogrammed cardiomyocyte to remain a cardiomyocyte in both identity and function is a topic that warrants further evaluation as new regenerative techniques are added to the toolbox. Along the same lines, it would be interesting to see if this OSKM-mediated reversion is cardiomyocyte-specific or if the approach can be used to generate other cell types such as neurons or hepatocytes. Because this de-differentiation reprogramming is unique from other reprogramming techniques in its use of generic reprogramming genes rather than cell-type specific genes, it would be intriguing to assess whether this transition to a proliferative immature state is unique to cardiomyocytes.

Another potential impact of this study is the ability to identify signaling pathways and genes that regulate the acquisition and silencing of the regenerative properties of immature cardiomyocytes. These genetic targets could be future focal points for manipulation in adult cardiomyocytes in order to increase their proliferation potential. Previous studies have shown that more targeted approaches for increasing adult cardiomyocyte proliferation have therapeutic promise. Several research groups have shown that members of the cyclin family of proteins are promising candidates (Mohamed et al. 2018; Pasumarthi et al. 2005; Shapiro et al. 2014). Other groups have evaluated proteins involved in the Hippo pathway as well as transcription factors like Gata4 and Meis1 (Mahmoud et al., 2013; Malek Mohammadi et al., 2017). All of these approaches have shown varying degrees of promise in allowing cardiomyocytes to enter a more proliferative state, thus they may benefit from application of knowledge obtained from OSKM-mediated reversible reprogramming in order to further hone in on specific pathways. Understanding the roadmap of de-differentiation during OSKM reprogramming can also be used to help other reprogramming methods to create more matured cardiomyocytes.

While there remains some interesting questions regarding this reversible OSKM-mediated reprogramming, this study provides foundational knowledge on the potential uses and limits of OSKM factors to modulate adult cardiomyocyte identity and achieve more regenerative cardiac tissue. OSKM-mediated identity reversion may, in the end, be a too powerful or non-specific methodology to modulate the maturation of cardiomyocytes, but the molecular pathways affected by this approach may ultimately be the key to unlocking the regenerative potential of adult cardiomyocytes and regenerating injured hearts

## Data Availability

Not applicable.
